# Biological and behavioral features and colonization of the sylvatic mosquito *Sabethes identicus* (Diptera: Culicidae)

**DOI:** 10.1371/journal.pone.0296289

**Published:** 2023-12-21

**Authors:** Maria Ignez Lima Bersot, Genilton Vieira, Jose Rodrigo De Moraes, Glauber Rocha Pereira, Monique Albuquerque Motta, Ricardo Lourenço-De-Oliveira

**Affiliations:** 1 Laboratório de Mosquitos Transmissores de Hematozoários, Instituto Oswaldo Cruz, Rio de Janeiro, Rio de Janeiro, Brazil; 2 Núcleo de Atividades de Extensão, Instituto Oswaldo Cruz, Rio de Janeiro, Rio de Janeiro, Brazil; 3 Instituto de Matemática e Estatística, Universidade Federal Fluminense, Niterói, Rio de Janeiro, Brazil; University of Cincinnati, UNITED STATES

## Abstract

Mosquitoes of the genus *Sabethes* are exclusively sylvatic species occurring in Central and South Americas, where they play a role in the transmission of arbovirus. S*abethes identicus*, a common bamboo-breeder species, has been found naturally infected with yellow fever virus. Our study aimed to describe biological and behavioral features of *Sa*. *identicus* in the laboratory, as well as establish and standardize an isolated colony for experimental assays. We tested different larval densities and evaluated larval and pupal development time, oviposition behavior, egg production, and longevity. We also shot and video-documented bionomics and behavioral aspects of the mosquitoes in the field and laboratory. A colony with more than 30 generations was achieved. Embryogenesis of *Sa*. *identicus* is completed in about three days with a 79% hatch rate, while larval and pupal development takes approximately 15–17 and nine days, respectively. The mosquito’s entire life cycle lasts approximately 30 days. Adult females could survive 71 days, and each individual laid an average of 88 eggs over their lifetime; 50% of females and males survived 37 and 24 days, respectively. Hematophagy peaks as early as the first week of emergence. The net content of a bamboo internode influenced the choice for oviposition, with the average number of eggs laid in those containing rearing water with larval and pupal exuviae being significantly higher than when they had only dechlorinated water or water with yeast. We documented for the first time an ecological association of weevils and *Sa*. *identicus*, where the oviposition of the latter depends on the weevil creating a hole in the bamboo wall for egg-laying purposes. Video recordings revealed for the first time the peculiar movements of gravid females during egg-throwing into tiny bamboo holes, as well as the laborious escape of newly emerged adults from the bamboo cavity, and mating behavior.

## Introduction

Yellow fever has a significant public health impact in both Africa and South America [1]. In the American continent, human infections have been only acquired in the sylvatic transmission cycle, where viremic non-human primates serve as the source of infection for arboreal mosquitoes. Of these, *Haemagogus janthinomys* Dyar, *Haemagogus leucocelaenus* (Dyar and Shannon), and *Sabethes chloropterus* (von Humboldt), have been identified as important main vectors [1–4]. Other *Sabethes* species [*Sa*. *albiprivus* Theobald, *Sa*. *belisarioi* Neiva, *Sa*. *cyaneus* (Fabricius), *Sa*. *glaucodaemon* (Dyar & Shannon), *Sa*. *soperi* Lane & Cerqueira and *Sa*. *identicus* Dyar & Knab] have also been found naturally infected with yellow fever virus (YFV) and other arboviruses (e.g., Triniti, Bussuquara, Mucambo, Murutucu, Mayaro, Kairi, Melao, Sororoca, Tucunduba, Xiburema) [5–9].

Despite their epidemiological relevance, the biology of *Sabethes* species has been poorly studied [2, 10–13]. Furthermore, knowledge of the behavior and most aspects of bionomics of *Sabethes* is even more limited. As far as we know, only two tree-hole *Sabethes* species have been colonized in the laboratory, both populations originated from Panamá: *Sa*. *chloropterus* [14–16] and *Sa*. *cyaneus* [17]. These colonies have favored the achievement of important information on their behavior and vector competence for arboviruses [14, 18]. Nonetheless, the genus *Sabethes* comprises 42 species found throughout Central and South America, and they exhibit variable habits and larval habitats in phytotelmata [19, 20].

The lack of isolated laboratory colonies has hindered progress in understanding the biology, behavior, and vector competence of *Sabethes* populations. In this study, we recorded and described some biological and behavioral features of *Sa*. *identicus* under experimental conditions and a few observations on the field. We aimed to establish, standardize, and optimize the maintenance of an isolated, perennial, and productive colony of this species. *Sabethes identicus* is a common sylvatic bamboo inhabiting mosquito species, which has been found in Argentina, Bolivia, Brazil, Colombia, French Guiana, Panama, and Costa Rica. It promptly bites humans in the forest [9, 21, 22] and has been recently found naturally infected with YFV [9].

## Materials and methods

### Fieldwork

Image recordings and mosquito collections were performed in two sites of the Atlantic Rainforest in the state of Rio de Janeiro, Southeastern Brazil: Reserva Biológica do Tinguá (RBT), in Nova Iguaçu (22°35’06.2"S 43°26’25.9"W), and Bom Jardim da Roça (BJR), in Duas Barras (22°04’53.2"S 42°35’41.4"W).

### Laboratory work

Laboratory breeding of *Sa*. *identicus* was started from 33 adults (18 females and 15 males) reared from eggs laid by females captured in RBT in 2013. In the laboratory, oviposition by field-collected females on wet filter paper or water was only achieved when removing one wing of gravid individuals 3–4 days after a blood meal on an anesthetized guinea pig. The immatures originating from these wild females were kept in rectangular plastic pans measuring 28.5x21.5x5.7cm containing ~1 liter of dechlorinated tap water supplemented with yeast powder, which was renewed every 2–3 days. Pupae were transferred to cylindrical dark pots (7x8cm) containing dechlorinated tap water, topped with a filter paper funnel whose narrow upper circular aperture (2cm in diameter) allows emerged adults to escape into a breeding cage. It was a wooden cage measuring 80x40x50cm, with a screen in front and at the back and glazed sides and top. Adults were fed daily with a 30% honey solution. Initially, we used one anesthetized guinea pig to serve as a source of blood for females. This was soon replaced with two to three anesthetized mice for 30 min, offered thrice weekly around noon. The receptacles for oviposition were perennially kept inside the cage and consisted of sections of field-collected bamboo internodes with tiny holes that had been naturally drilled by weevils and partially filled with dechlorinated tap water. The upper node was removed to facilitate manipulation, and the top of the internode was sealed with a cotton gauze stopper. A small number of shrub branches served as supplementary adult resting places in the cage. All stages were maintained in an insectary at 25–26°C, 80 ± 10% RH, with a 12 h:12 h light:dark cycle.

Once an expressive number of *Sa*. *identicus* adults were attained, and a set of experiments, observations, and video recordings were conducted to describe some of their biological and behavioral features in the laboratory. Each experiment was conducted twice.

To evaluate the pupal stage duration, 150 one-day pupae were split into pots holding 35 or 40 individuals of each gender. Pairs of pots were transferred to four cages and observed daily until the adults emerged. The number and gender of emerged adults were recorded daily.

In order to assess feeding behavior, egg production, and longevity, groups of 35 three-day-old adults of each gender were kept in four cages, each containing three bamboo internodes naturally pierced by beetles. Each bamboo internode contained different liquids as choices for oviposition, as described below. Blood meals consisting of two anesthetized mice were simultaneously offered in each cage on alternating days from midday to 2 p.m. when the number of blood-fed females in each cage was immediately recorded. As the number of daily blood-fed females per cage was usually low, we grouped the outcomes into seven periods (P1 to P7) of nine days each. Dead mosquitoes were removed daily from the cages and counted according to gender.

To evaluate the influence of bamboo content in attracting oviposition, three bamboo sections of similar dimensions and with an equal number of holes were simultaneously offered in two cages (G1 and G2): Bamboo 1 contained 65 ml of water that had previously been used for rearing immature forms of *Sa*. *identicus* containing co-specific larval and pupal exuviae (RW); Bamboo 2 contained 65 ml of dechlorinated tap water with 0.1g of yeast (YW), and Bamboo 3 contained 65 ml of dechlorinated tap water (DW). The bamboo contents were removed every other day to count the eggs, washed internally with tap water, refilled with the same type of liquid (RW, YW, or DW), and then returned to their respective cages. The position of bamboo sections inside the cage was changed after each egg counting session.

The eggs that were obtained every second day were transferred to plastic pans, as described above. To assess the embryonic development time and egg viability, the number of seeded eggs per pan was recorded, and the newly hatched larvae in each pan were counted daily and relocated into another pan for rearing.

To evaluate larval development, larvae hatched on the same day were distributed in groups of pans as follows: (a) three pans with 30 larvae containing 0.1g of yeast (pans 1 to 3); (b) three pans with 60 larvae containing 0.2g of yeast (pans 4 to 6); and (c) three pans with 90 larvae containing 0.3g of yeast (pans 7 to 9). Replicates were made with the three groups of pans containing the different larval densities (30, 60, and 90 larvae/pan) submitted to two conditions of luminosity: (a) 12 h:12 h light:dark cycle and (b) 24 dark, except during larval counting. Larvae were counted every other day from the fifth day of hatching when the rearing solution containing the three different amounts of yeast was changed. Comparisons were made between pans containing the same larval densities and different luminosity conditions.

Some behavioral features of *Sa*. *identicus*, such as mating, egg-laying, and scape of adults from pierced bamboo, were recorded with a Phantom® Miro 310 one-megapixel camera that can record up to 3,200 frames-per-second at full 1280 x 800 resolution. In addition, special lenses (IF-series, KC VideoMax, Infinity®) suitable for taking high-resolution photographs from short distances were used. Video recordings were made in a cubical glass cage (40cm) containing pierced bamboo stalks, where 20 *Sa*. *identicus* males and non-gravid females aged 3–10 days had been released there two days before. Egg-laying behavior was filmed in a distinct assay with 30 *Sa*. *identicus* gravid females, aged between 7 and 15 days, who had been released into the cubical cage 1–2 days before. One day before filming the escape of newly emerged adults from the bamboo internode, 20 pupae, aged 8–9 days, were put inside a bamboo stalk that had been naturally pierced by a weevil containing dechlorinate tap water and sealed at the top. Photographs of weevils in nature were taken with a Nikkormat 35mm camera, with micro-Nikkor 1:3.5-f55 lenses.

## Statistical analysis

To assess the probability that pupal development would be completed in nine days instead of eight according to gender (female, male), a logistic regression model was adopted. From this model, the odds ratios (OR) and their respective 95% confidence intervals (CI) were estimated. To assess the significance of the effects of gender and cage on the chance of completing the pupal development time on the ninth day, the Wald test was used with a significance level of 5%. To analyze the average number of females that took a blood meal in seven periods (P1 –P7) over their lifetime, we used Friedman’s non-parametric test, followed by Siegel-Castellan’s multiple comparisons test (post-hoc Test), with Bonferroni correction [23]. To compare the number of eggs laid in bamboo internodes containing different liquids as choices for oviposition (RW, YW, and DW in bamboos 1, 2, and 3, respectively), we used the multilevel Quasi-Poisson regression model with random intercept containing two hierarchical levels. The repeated measurements of the number of eggs (counts) were considered the 1^st^ level units, and the cages the 2^nd^ level units (group). The number of eggs (outcome) collected from each bamboo placed inside each cage was recorded on multiple occasions. We could then estimate the average ratios (AR) from this multilevel mode; their 95% confidence intervals (CI) and p-values of Wald’s significance test were also calculated. The variance partition coefficient (VPC) was also calculated. This is a quantitative measure between 0% and 100%, which provides information on the proportion of total variation of the outcome that can be attributed to differences between the groups. The multinomial logistic model was also adopted to assess the association of the variables "larval density" (30, 60 and 90 larvae/pan) and "luminosity condition" (12 h:12 h light:dark cycle and 24h dark) with the period of time in which the larvae turned into pupae. For this analysis, the outcome was considered polytomous, with four classes of larval development times: 15 to 17 days (baseline category), 18 to 19 days, 20 to 21 days, and 22 to 40 days. Odds ratios (OR), with 95% confidence intervals (CI), were also produced based on this model. Statistical analysis was performed using the RStudio program, version 4.2.2 [24]. For the application of non-parametric statistical tests, the PMCMRplus package was used [25]. The logistic regression model was fitted using the “glm” function from the R base package, while the multilevel quasi-Poisson regression model was fitted using the “glmmPQL” function from the MASS package of the RStudio [26]. To fit the multinomial model, the “vglm” command from the VGAM package was used [27].

### Ethical statements

Mosquito feeding on mice was licensed by the Ethics Committee on Animal Use–CEUA, Instituto Oswaldo Cruz (LW-32/14). Mosquito collection at Reserva Biológica do Tinguá was approved by local environmental authorities: Sistema de Autorização e informação em Biodiversidade (SISBIO), Brazilian Ministry of Environment (license # 37362–2). This study did not include endangered or protected species.

## Results

**Fig 1:**
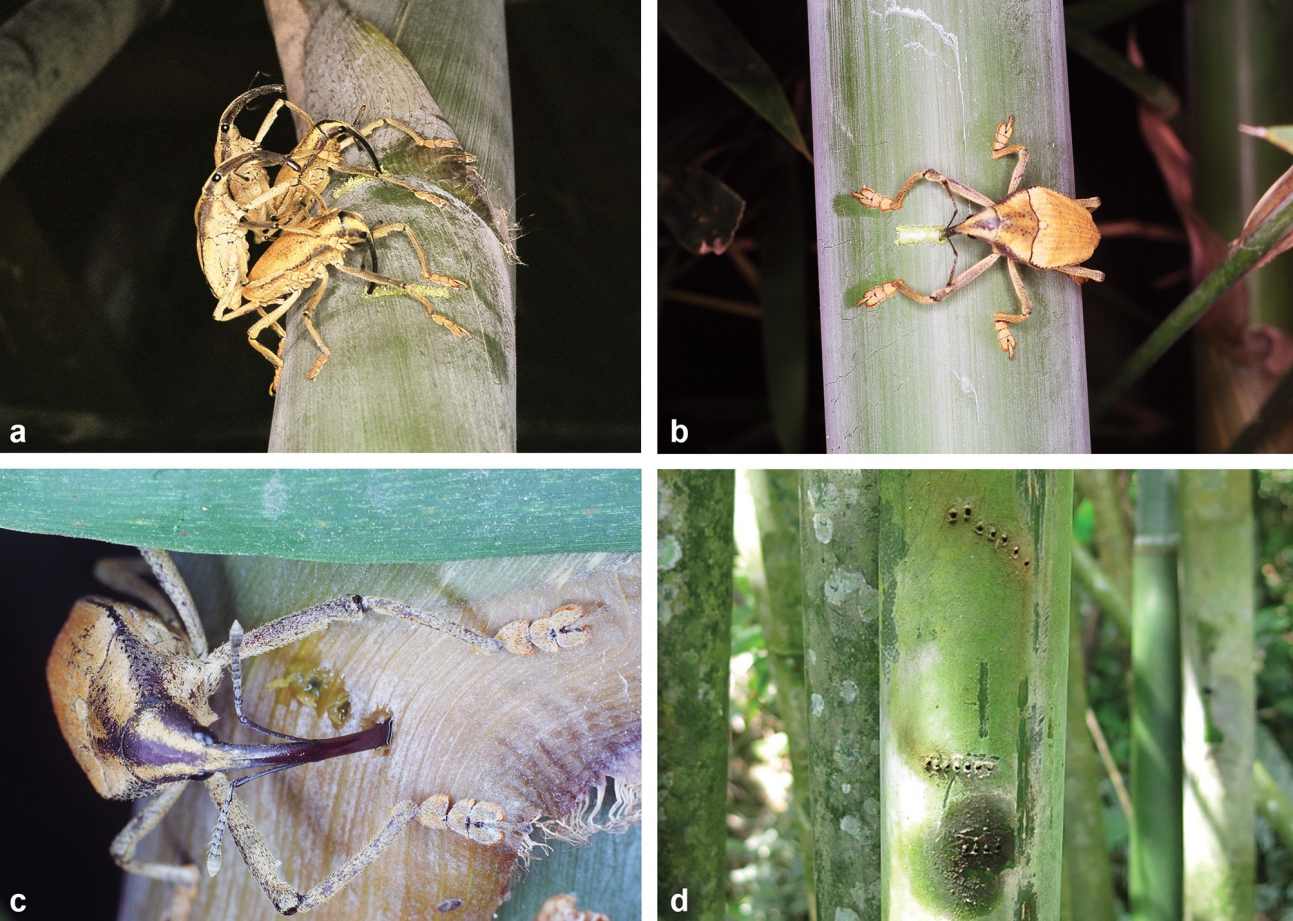
Bamboo internodes pierced by weevils, the larval habitats of *Sabethes identicus* in nature. Females of *Rhinastus sternicornis* perforating bamboos while mating (A) or lonely (B, C). A bamboo internode displaying three roughly linear series perforations (D) in a bamboo grove in the Atlantic Forest fragment at Bom Jardim da Roça, Duas Barras county, Rio de Janeiro, Brazil.

During the fieldwork, immature forms of *Sa*. *identicus* were exclusively found in bamboo internodes containing tiny perforations previously made by weevils for oviposition. In contrast, this mosquito’s immature form was never found in cut, broken, cracked bamboo or in those presenting a large hole, even when it was as narrow as 5–7mm in diameter (such as, for example, the exit hole made by the weevil to escape from the bamboo internode cavity after completing its development). At BJR, immature stages of *Sa*. *identicus* were found only in bamboos pierced by *Rhinastus sternicornis* (Germar) and *Astyage lineigera* Pascoe (Coleoptera, Curculionidae) females (Fig 1). These weevil species usually make a roughly linear series of around six (3–9) perforations in a bamboo grove to oviposit, which are later used by *Sa*. *identicus* to throw eggs into the internode cavity.

In the laboratory, the duration of the pupal development at 25–26°C was 8–9 days (39% and 61% of adults emerged in eight and nine days, respectively) (S1 Table). In total, 38% of males and 40% of females emerged in 8 days and 62% of males and 60% of females emerged in 9 days. The chance of completing the pupal development in nine instead of eight days was 1.459 and 1.632 in females and males, respectively (Table 1). However, there was no significant difference in the chance of completing the pupal development in nine instead of eight days between males and females (OR = 1.118, *p* = 0.636).

**Table 1:** Odds ratios (OR) of *Sabethes identicus* to complete pupal development in 9 days instead of 8 days.

Variable	Outcome (%)		Logistic model results		
	8 days (N = 118)	9 days (N = 182)	OR	CI 95%	*p*-value *(Wald)*
Intercept			1.459	(1.053–2.021)	0.023
**Gender**					
Male (n = 150)	38.0%	62.0%	1.118	(0.703–1.778)	0.636
Female (n = 150)	40.7%	59.3%	1	-	-

**Fig 2:**
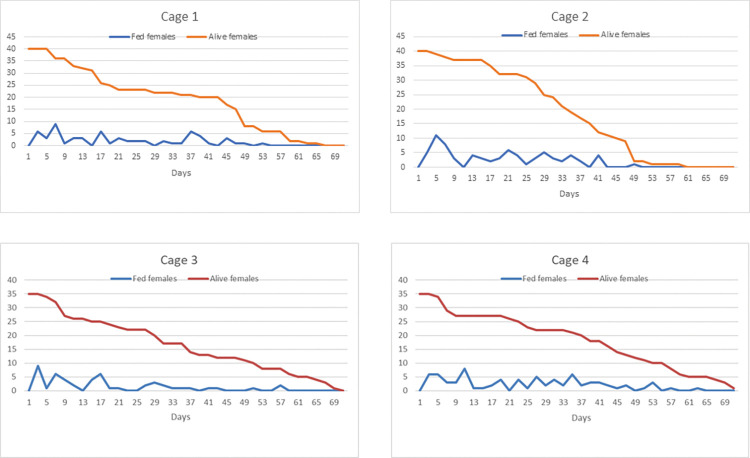
Number of blood fed and alive *Sabethes identicus* females in four cages.

The biting activity profile was similar for female mosquitoes regardless of the cage they were kept in with a slight biting peak between days 3 and 11 after emergence from pupa (P1). There was a statistically significant decrease in biting activity from days 53 to 71 (Table 2 and Fig 2). A significant effect was found of lifetime on the number of fed females, with a significant difference being found between the number of females taking blood meals during at least one pair period (P6 and P7). The Siegel-Castellan’s multiple comparisons test, with Bonferroni correction, revealed a difference in the mean number of females taking blood meals at P1 and P6 (p = 0.022) and P1 and P7 (p = 0.003). No difference was found between other pairs of periods (p> 0.05).

**Table 2:** Mean number of *Sabethes identicus* females that took a blood at seven day intervals (P1 to P7) throughout their lifetime.

	Intervals (days)							
Cage	P1 3 to 11	P2 13 to 21	P3 23 to 31	P4 33 to 41	P5 43 to 51	P6 53 to 61	P7 63 to 71	p-value*
G1	4,4	2,6	1,6	2,6	1,0	0,2	0,0	<0,001
G2	5,4	3,6	3,2	2,4	0,2	0,0	0,0	
G3	4,4	2,4	1,4	0,8	0,4	0,4	0,0	
G4	5,2	1,6	3,2	3,2	1,2	0,8	0,2	

*Friedman nonparametric test.

Eggs were found in bamboos containing the three types of liquid offered for oviposition (RW, YW, and DW), although the number of eggs found in each varied (Table 3). The total average number of eggs recovered from bamboo containing RW was 53.8% higher than that of bamboos filled out with YW (AR = 1 / 0.650 = 1.538; p-value = 0.048) and 56.5% higher than that of bamboos containing DW (AR = 1/ 0.639 = 1.565; p-value = 0.041). No significant difference was found in the average number of eggs in bamboos containing YW and DW (AR = 0.650/0.639 = 1.017, CI 95% = 0.636–1.629). From the variance partition coefficient (VPC), it was observed for the null model (without considering bamboo contents) that 12.6% of the variation in the number of eggs was attributed to differences between cages. With the inclusion of bamboo content, the VPC value increased slightly to 12.8% (Table 3).

**Table 3:** Estimated average ratio of number of eggs laid by *Sabethes identicus* in bamboos containing three types of liquid as choices for oviposition: Rearing water (RW), dechlorinate water with yeast (YW) and only dechlorinate water (DW), considering multilevel modeling.

Characteristic	Number of eggs	Null model			Model including variable “bamboo content”		
		AR	CI 95%	p-value (*Wald*)	AR	CI 95%	p-value (*Wald*)
**Fixed part**							
Intercept		26.940	(16.401–44.252)	<0.001	35.283	(20.653–60.277)	<0.001
***Bamboo content***							
RW	5411				1	-	-
YW	3516				0.650	(0.425–0.994)	0.048
DW	3456				0.639	(0.416–0.980)	0.041
**Random part**							
Cages		0.2163			0.2176		
VPC		12.6%			12.8%		

AR = Average Ratio; VPC = Variance partition coefficient

Outcome: Number of eggs; Explanatory variable: Bamboo content

Group: 4 cages [420 observations: 35 observations for each combination cage (G1, G2, G3, G4) x bamboo content (RW, YW, DW)]. Total number of eggs per cage: G1 = 3260; G2 = 5404; G3 = 1284; G4 = 2435.

**Fig 3:**
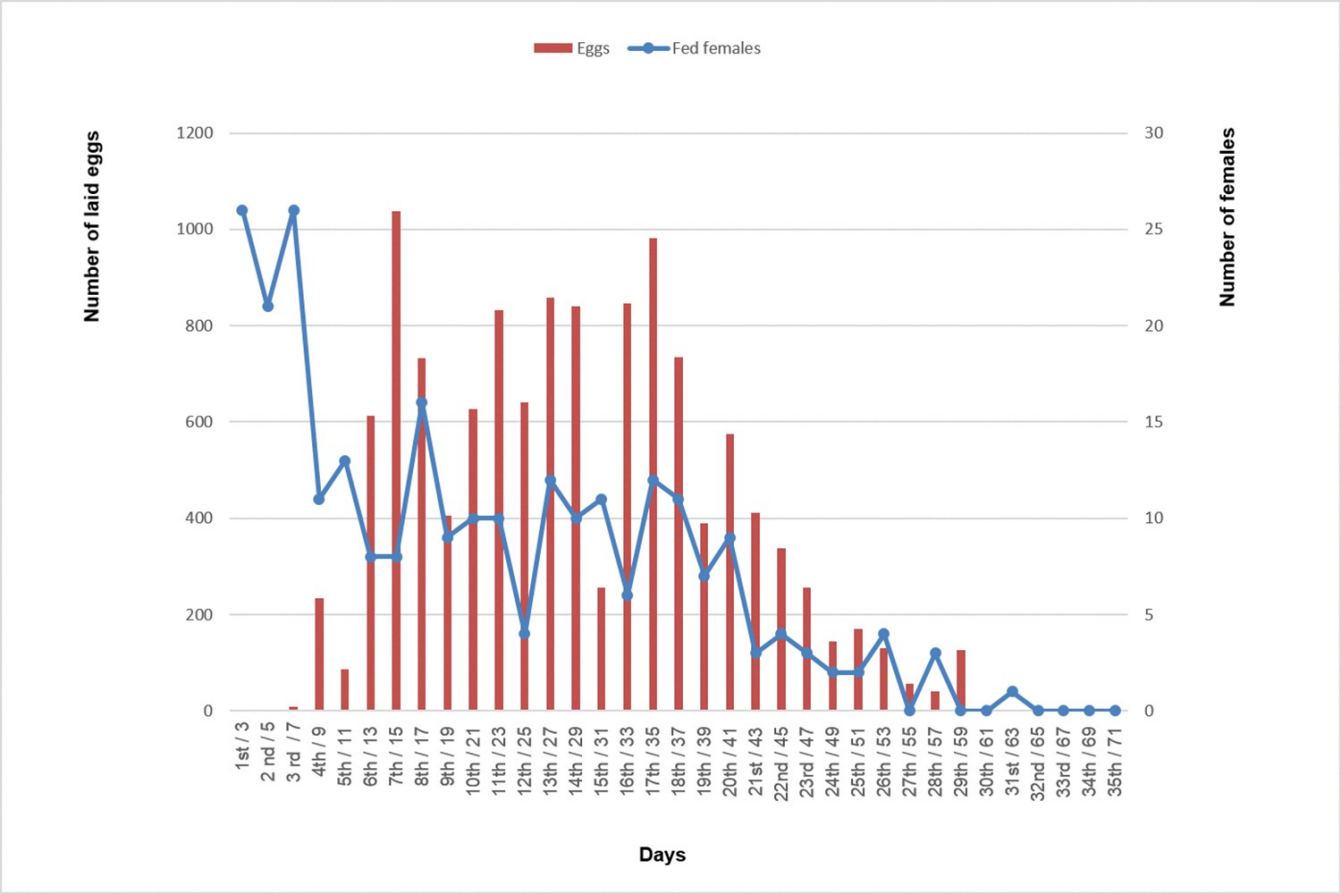
Number of laid eggs and blood fed females of *Sabethes identicus* over their lifetime. On the abscissa (x-axis), the cardinal and ordinal numbers refer respectively to the blood meal taken by the females and the number of days elapsed from their emergence from pupa.

Considering the total daily number of eggs collected from the four cages, the period of greatest productivity was between days 13 and 41 post-emergence, with a peak on day 15 and another around day 35 (Fig 3).

**Fig 4:**
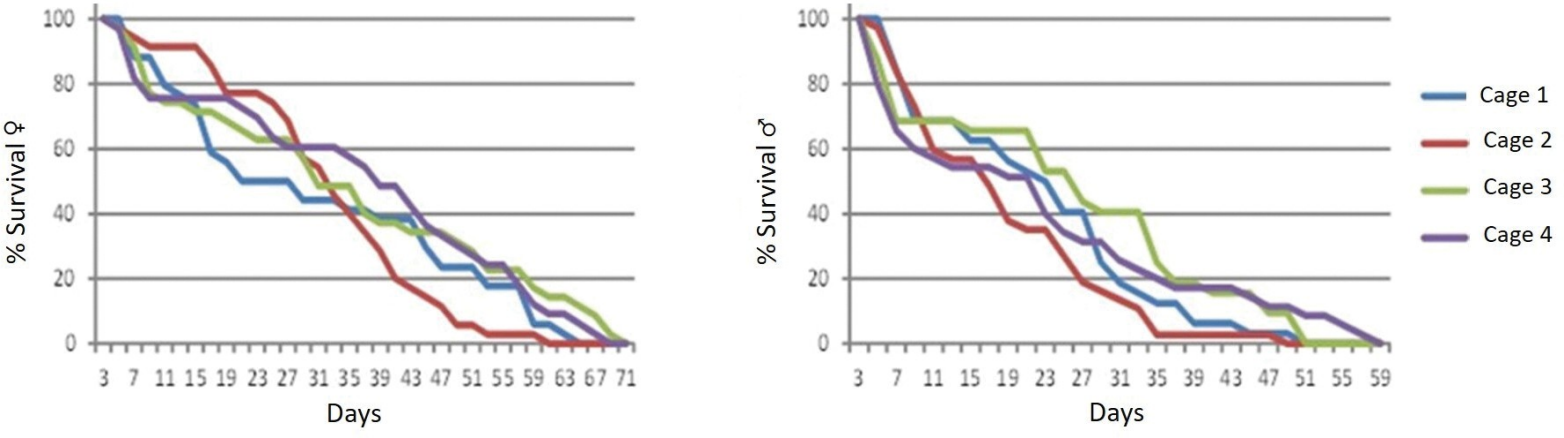
Percentage of surviving females and males of *Sabethes identicus* in four cages.

The adults of *Sa*. *identicus* lived no more than 71 days under the described laboratory conditions, with females surviving longer than males (Fig 4). The pattern of daily mortality was similar in the four cages. The majority (around 80%) of females survived approximately 11 days in the G1, nine days in the G3, seven days in the G4, and 18 days in the G2, with an average survival of 10 days. Approximately 80% of males survived seven days post-emergence in the G1 and G2, six days in the G3, and five days in the G4, with a total average of six days. Overall, approximately 50% of females and males survived until days 37 and 24, respectively.

**Fig 5:**
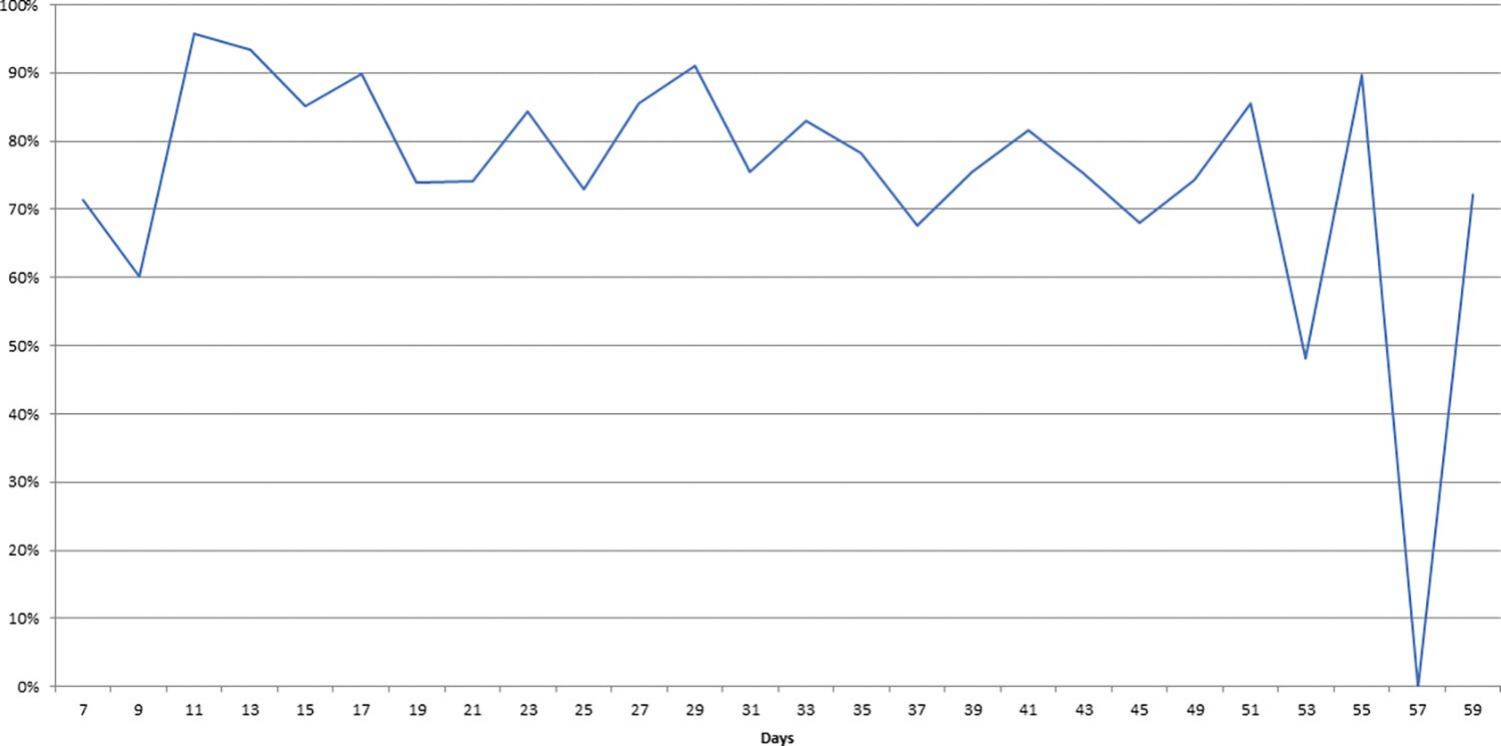
Percentage of eggs hatched laid by *Sabethes identicus* females over their lifetime.

Approximately 90% of eggs completed embryonic development in 2–4 days, with only a few eggs hatching on days 5–9 (Table 4). The viability rate of eggs ranged from about 98% to 50%, with a higher rate of hatchings in oviposition made at the beginning of the female’s lifespan (until ~30 days of emergence) (Fig 5).

**Table 4:** Number and percentage of daily hatched larvae of *Sa*. *identicus* in two laboratory assays.

Assay	Days								Total
	1 to 2	2 to 3	3 to 4	4 to 5	5 to 6	6 to 7	7 to 8	8 to 9	
1	119	1648	992	110	28	5	7	1	2910
2	165	811	461	40	18	2	2	1	1500
Total	284	2459	1453	150	46	7	9	2	4410
**%**	**6.43**	**55.76**	**32.94**	**3.40**	**1.04**	**0.15**	**0.20**	**0.04**	**100 **

**Fig 6:**
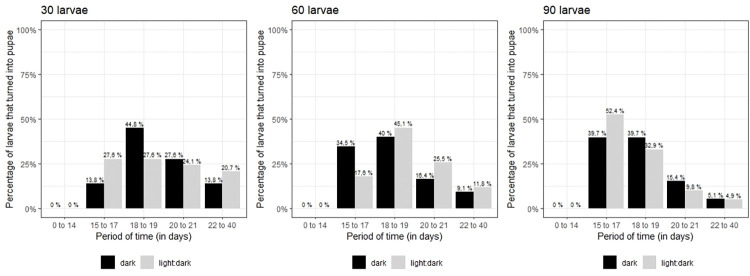
Percentage of larvae of *Sabethes identicus* turning into pupae in five day periods, submitted to two luminosity conditions: 12h:12h light:dark cycle and 24h dark.

Most *Sa*. *identicus* larvae (90%) became pupa between the 15^th^ and the 21^st^ day after egg hatching, although larval development until larval-pupal metamorphosis varied according to larval densities (Fig 6). Nearly 79% and 86% of larvae reared in pans containing 30 individuals became pupa in this period when exposed to 12h:12h light:dark cycle and 24h dark, respectively. In this same period, 88.2% and 90.9% of larvae became pupa in pans with 60 individuals respectively exposed to the light:dark cycle and only in the darkness, while the respective figures for pans with 90 larvae in this period were 95.1% and 94.8%. There was no statistically significant association between the luminosity condition of rearing and the time for larval-pupal metamorphosis regardless larval densities (S2 Table). However, the larval density showed statistical significant association with the time for larvae to turn into pupae, with a reduction in the chance of occurring larval-pupal metamorphosis in the three day periods considered from day 18 (18–19, 20–21 and 22–40 days) compared to 15–17 days as the larval density increases. Accordingly, the chance of larvae to turn into pupae in 18–19, 20–21 and 20–40 days versus 15–17 days was respectively 2.2, 4.6 and 7.7 times greater when the larval density was 30 individuals/pan compared to 90 larvae/pan. Similarly, the chance of the occurring larval-pupal metamorphosis in the analyzed day periods from day 18 versus 15–17 days was respectively 2, 2.9 and 3.6 times greater when the density was 60 larvae/pan compared to 90 larvae/pan.

**Fig 7:**
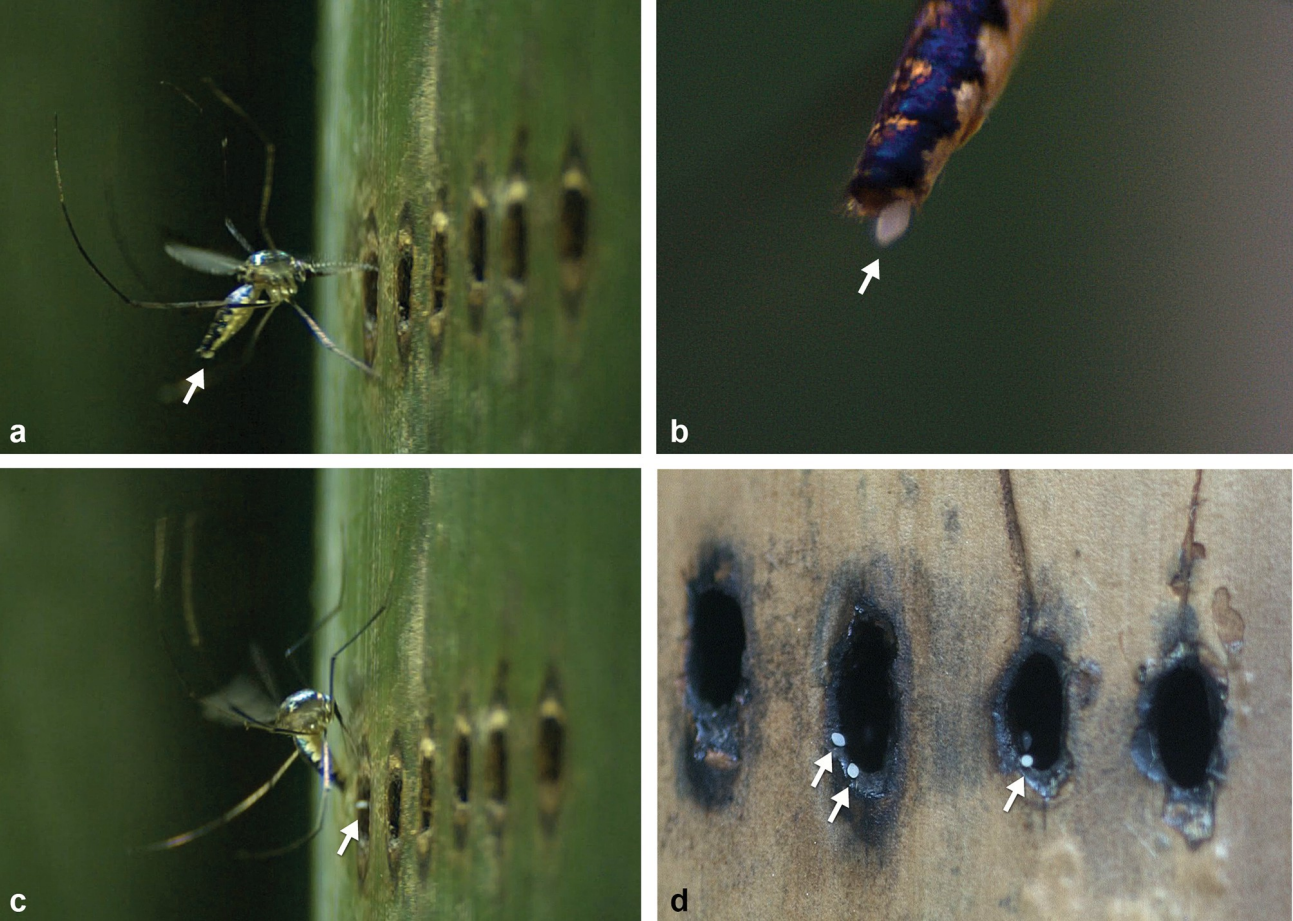
Frames taken from *Sabethes identicus* gravid female laying egg in the laboratory, filmed at 3200 frames per second. The recording illustrates the body and leg positions assumed before (A) and during (C) egg-throwing into the bamboo internode cavity through a tiny perforation drilled by a weevil. The arrows highlight the egg still held at the abdomen tip (A, B) and traveling toward the perforation in the bamboo internode (B). Some females probably missed the lumen of the aperture and the egg failed to reach the internode cavity. The eggs are stuck in the edge of the hole (D).

Video images and selected frames from edited video recordings illustrate some steps of the distinctive egg-laying behavior of *Sa*. *identicus* into pierced bamboo (Fig 7 and S1 Video). Accordingly, the gravid female approaches one of the thin openings in the wall of the perforated bamboo and performs a series of rapid, short, back-and-forth, and up-and-down flights in front of it as if it were gliding in the air. At this moment, the egg to be launched is already visible at the tip of its abdomen (Fig 7A and 7B). The proboscis makes longitudinal movements until it becomes essentially straight. That is when the female points the tip of the proboscis to the upper edge of the opening and seems to be almost ready to throw the egg (Fig 7A and S1 Video). At this stage the following steps are noted (S1 Video): the proboscis is forced against the perforation and the wall of the bamboo itself and it bends down; the female performs a short upward flight, strongly thrusts its entire abdomen forward, moves its mid-legs backward and downward, while moving the fore- and hindlegs forward; the fore-legs hit the bamboo outer wall vigorously; the egg is thrown forcefully through the opening (Fig 7C and S1 Video). Then, the female’s body starts unbending and it flies back, resuming the typical hovering flight (S1 Video). The female may immediately and repeatedly return to the same opening and go through the same motions to consecutively throw other eggs (S1 Video). Some eggs fail to be thrown inside the bamboo internode, remaining on the edge of the opening (Fig 7D).

In nature, the entire development of immature stages until the emergence of adults of *Sa*. *albiprivus* takes place in a gloomy atmosphere inside the bamboo internode, whose tiny holes drilled by a weevil are the only accesses for newly emerged adults to reach the external environment. We video-recorded the remarkable behavior of newly emerged adults to escape from the bamboo cavity in the laboratory (S2 Video). The adult forelegs first cross the hole in the bamboo wall. It excitedly kicks its forelegs and vigorously taps the outer surface of the bamboo several times, which apparently helps to propel it forward. After a short while, the head becomes visible in the opening lumen or may be partially exposed. At this point, with its forelegs laying on the outer surface of the bamboo, the adult produces rapid movements that gradually propel its body forward and outward. Immediately after the first propulsions, the entire head and anterior portion of the thorax are usually exposed. The first propulsions seem to be the most difficult to perform. The remainder of the thorax is exposed with one to four subsequent thrusts, but the mid- and hindlegs are not yet exposed by this stage. They will come out together with the abdomen in an essentially single movement that culminates in immediate flight. Once the insect puts its forelegs out of the opening, it rarely takes breaks between the propulsions to escape from the bamboo. Indeed, the adults rarely rest on the outer wall of the bamboo after it escapes the cavity, suggesting that when they leave the cavity, they are already ready to fly. Although it is less common, the mosquito sometimes comes out upside down. Despite the position used to escape from the cavity, some individuals seem to have more difficulty passing through the orifice, waving their forelegs numerous times and for a longer period of time. Eventually, they all succeed in leaving the cavity.

When making video records of the mating behavior of *Sa*. *identicus* (S3 Video), we remarked that courtship and copulation always occur with females resting on vertical or inclined branches of shrubs at various angles or the cage walls and screen. These surfaces are frequently patrolled by males in the search for potential mates. Males do not seize females in midair but fly up and down these resting sites until they find a female and rapidly hover behind her. At this point, the male bends the tarsi of the right mid-leg into the shape of a hook with which they try to seize the dorsal surface of the resting female’s wing. Still seizing the female’s wing, the male quickly rolls upside-down and lands on the same substrate where the female is, but in an inverted position so that the ventral surfaces of their bodies face each other. Non-receptive females may flick the hindlegs, causing the approaching hovering males and even those that are attempting to, or who have already managed to seize the upper surface of their wing to go away (S4 Video). When aligned in this position, the male pushes its entire body upward, followed by the tip of the abdomen so that they make genital contact (S3 Video). Coupling is achieved usually after a few attempts to make superficial contact between the genitals. Only 1–3 seconds after the male’s genitalia is locked onto the female’s, the male removes the hooked mid-tarsi that seized the dorsal surface of his mate’s wing. Immediately, his right and left mid-legs initiate a series of oscillatory alternate or synchronous movements towards (inward) the female and away (outward) from her. These mid-leg motions occur at the same time as the vigorous downward flicking of the proboscis. This gives the impression that the proboscis and antenna are rapidly vibrating together. Although distinct, the movements of mid-legs and proboscis-antenna are rhythmically cyclical and vary in frequencies during copulation but last throughout it. Eventually, coupled male and females move their bodies together towards and away from the resting surface on which they remain grabbed. Except for the frantic and repetitive motions performed by the male and the slow forward and backward movements of the female’s hindlegs, the couple remains essentially still throughout copulation, which may last between nine minutes and around one hour. During this time, their wings remain motionless. While the male keeps his wings fully open in an axis transverse to the thorax, the female’s wings remain closed, with one placed over the other in line with the abdomen. Both the female and male may start the release. When the male starts the release, it slowly separates the genitals and may fly away on his own volition, while the inseminated female remains grabbed on the same resting surface. Alternatively, the female kicks the male with its hindlegs, causing the mate to rapidly separate the genitals and leave.

## Discussion

The laboratory experiments, field and laboratory observations, and video documentations we conducted provided totally original information on the biology and behavior of *Sa*. *identicus*. Similar information on other *Sabethes* species and other Sabethini genera is limited. Moreover, comparing these few previous information with our data is difficult, as the environmental conditions and methods used for obtaining the information were quite heterogeneous. For instance, the pupal stage lasted longer (8–9 days) for *Sa*. *identicus* kept at 25–26°C than for *Sa*. *chloropterus* (5–6 days) colonized within a larger temperature range (23–30°C) [16]. This temperature variation may have accelerated metamorphosis. Indeed, the pupal development in laboratory colonies of *Sa*. *cyaneus* and *Sa*. *aurescens* (Lutz) maintained at 27±1°C and 4–33°C lasted 6–8 and 6–10 days respectively [17, 28].

In contrast to previous reports on *Sa*. *chloropterus* females not taking a bloodmeal before the 5^th^ day of emergence in the laboratory [16], *Sa*. *identicus* promptly fed on mice from the 3^rd^ day. Furthermore, while the number of blood-fed females peaked at around the first week after emergence in *Sa*. *identicus*, this was reported to occur during the 3^rd^ and 4^th^ weeks in *Sa*. *chloropterus* [16]. The time to complete embryonic development in *Sa*. *identicus* was the same as that reported for *Sa*. *cyaneus* [17], with egg hatching peaking 2–4 days after oviposition, which is also very similar for *Sa*. *chloropterus* (3–4 days) [16]. Most larvae transformed into pupae between the 17^th^ and 19^th^ day after hatching in *Sa*. *identicus*, while a shorter period (12–16 days) was reported for *Sa*. *chloropterus* [16]. As well as specific species characteristics, variances in larval density, availability of food in the rearing water, and temperature would help to explain these differences.

As for the adult stages, the maximum longevity of females and males of *Sa*. *identicus* was 71 and 59 days, respectively, much less than the 140 days previously reported for females of *Sa*. *chloropterus* [16]. On the other hand, 50% of *Sa*. *identicus* females survived approximately 37 days after the pupa’s emergence, similar to *Sa*. *chloropterus* [16], where the average longevity ranged from 32.5 to 47 days.

The development time of *Sa*. *identicus* under the laboratory conditions used in this study ranged from 27 to 49 days from egg hatching to adult emergence. This is in line with previous findings on two cogeneric species: *Sa*. *chloropterus* (18–50 days) and *Sa*. *cyaneus* (20–55 days) [11].

No difference was detected in the average number of eggs obtained in bamboo containing only DW and YW. However, the bamboo containing water that had previously been used for rearing immature forms of *Sa*. *identicus*, with co-specific larval and pupal exuviae, seemed more attractive to females to oviposit, as the average number of eggs recovered in bamboos with RW was significantly higher than in bamboos with DW and YW.

Larvae reared in pans with a higher larval density (90 larvae) changed to pupae more rapidly than in those containing 30 and 60 larvae, regardless of whether they were kept in a 12 h:12 h light:dark cycle or in the darkness, as would occur inside the cavity of the bamboo internode, their natural habitat. Cannibalism has been reported to happen in *Sa*. *identicus*, mostly until the 5^th^ day of larval development, when most individuals are on the 2^nd^ larval instar, and around 10% of individuals may be killed [29].

Taking these data together, we obtained an enduring and productive colony of *Sa*. *identicus* rearing 90 larvae per pan containing ~1 liter of dechlorinated tap water supplemented with 0.3g yeast powder renewed every 2–3 days and submitted to a 12 h:12 h light:dark cycle. Initially, the oviposition locations consisted of sections of bamboo stalks that had naturally been pierced by weevils, as described further above. As the search for these pierced bamboos in the forest was laborious and cut internodes deteriorated over time in the laboratory, the bamboo stalks were substituted with success by pieces of delicately multi-pierced PVC pipes with both ends sealed by rubber plugs containing only dechlorinated tap water as oviposition choice.

Following essentially the same criteria, we established a laboratory colony of *Sa*. *albiprivus* [30]. The two colonies we obtained allowed us to evaluate the vector competence of *Sa*. *albiprivus* and *Sa*. *identicus* for Zika virus and YFV (wide-type and 17DD attenuated vaccine virus) [31–33]. They also allowed us to use video recordings of some of their unique behaviors in the laboratory, such as the oviposition of *Sa*. *albiprivus* in sapucaia nuts simulating a tree hole with a small entrance that is its larval habitat in the wild [30]. Here, we recorded and described for the first time the oviposition into a pierced bamboo internode cavity and the escape of newly emerged adults from it, as well as the mating behavior of *Sa*. *identicus*.

The mating behaviors of other Sabethini, including that of the cogeneric species such as *Sa*. *chloropterus* and *Sa*. *cyaneus* have been described and illustrated with special attention to the complex male courtship behavior [16, 17, 34–39]. However, no video recordings are available. Here, we reported and obtained video records of the copulatory behavior of *Sa*. *identicus*. Overall, it is essentially the same previously described in detail and richly illustrated with drawings for *Sa*. *chloropterus* [37], except that the female always affects the release after copulation by kicking the male with its hind legs in the later species. We demonstrated that release may also be made by the male in *Sa*. *identicus*, as has been described for *Sa*. *cyaneus* [17].

The egg-throwing behavior and egg-laying in phytotelmata while in flight have been reported for some Sabethini [16, 34, 40]. Nonetheless, the video recording of the unusual mechanism of egg-throwing behavior, as described herein, has been available only recently for *Sa*. *albiprivus* [30]. The difference between *Sa*. *albiprivus* and Sa. *chloropterus*, who throw eggs through the narrow entrance of tree holes [16, 30], and *Sa*. *identicus* is that *Sa*. *identicus* throw their eggs via minuscule perforations previously drilled by weevils in bamboo internodes. This requires the gravid female species to perform some precise movements for the egg to be thrown successfully into the cavity while in flight. While the position of legs and general movements performed by *Sa*. *identicus* during egg-laying are essentially the same as previously recorded for *Sa*. *albiprivus* and *Sa*. *chloropterus*, the narrowness of the holes in the bamboo wall requires the *Sa*. *identicus* female to get very close to them, first with the tip of the proboscis, which is usually introduced at the edge of the hole, culminating in the collision of the dorsal surface of the proboscis (which with the head bent downwards) and the tarsi of forelegs on the outer wall of the internode at the time of throwing the egg. The female of the mosquito *Topomyia yabarensis* Miyagi also performs egg-throwing to oviposit into the cavity of bamboo internode through tiny holes drilled by cerambycid beetles. To do this, they approach the hole in flight, grasp the outer surface of the bamboo with the fore- and mid-tarsi, release their fore-legs, positioning them upwards, and throw the egg while still grasping the internode with the mid-tarsi and continuing to flap its wings [34]. This performance differs from that of *Sabethes* species, whether they breed in bamboo or tree holes, like *Sa*. *identicus*, *Sa*. *albiprivus* and *Sa*. *chloropterus*, as their mid-legs remain free and directed downward and outward. They may even hit the outer wall surface of the phytotelmata with the forelegs, but they do not grasp it, laying the egg while in flight.

Immature forms of mosquitoes other than *Sabethes* develop in bamboo pierced by beetles, such as *Wyeomyia* of the subgenus *Miamyia*, but the escape behavior of newly emerged adults from the internode cavity has not yet been documented. It has been described that tephritid flies (Family Dacinae) escape from the bamboo cavity through the larger exit hole made by the weevil [41], not through the tiny egg-laying puncture drilled by the beetle, as recorded by our videos for *Sa*. *identicus*. Although some individuals took a little longer than others to escape, all *Sa*. *identicus* emerged inside the bamboo cavity and managed to get out through any of the tiny holes, indicating an adaptation to this type of habitat. In the colony we established, we successfully used dark pots topped with a filter paper funnel with a narrow upper circular aperture through which the adults emerged into the cage.

Overall, our experimental data and video records allowed us to produce a standardized and optimized thriving colony of *Sa*. *identicus*, allowing us to contribute to the literature on the biology and behavior of *Sabethes*, a genus comprising several arbovirus vector species.

## Supporting information

S1 Table Daily number and percentage of emerged adults of *Sabethes identicus* in the laboratory. (DOCX)

S2 Table Odds ratio (OR) for larva to turn into pupa in three day periods versus 15 to 17 days (baseline), according to larval density (30, 60 and 90 larvae/pan) and the luminosity condition of rearing (12 h:12 h light:dark cycle and 24h dark). (DOCX)

S1 Video Gravid females of *Sabethes identicus* laying eggs into the bamboo internode cavity through perforations drilled by weevil, filmed at normal speed (~30 frames per second) and 3,200 frames per second in the laboratory. The sequences show the rapid and short up and down flights performed by the female prior to point the tip of the proboscis to the upper edge of the perforation, thrust the abdomen and throw the egg. Notice the egg to be thrown held at the tip of the abdomen.
(MP4)

S2 Video Video recording at normal speed (~30 frames per second) of newly emerged adults of *Sabethes identicus* escaping from the bamboo internode cavity through tiny perforation drilled by a weevil in the laboratory. (MP4)

S3 Video Courtship and copulation of *Sabethes identicus*. Two sequences filmed at normal speed (~30 frames per second) in the laboratory.
(MP4)

S4 Video Non-receptive females of *Sabethes identicus* may reject to be copulated by flicking the hindlegs causing the males to go away, even those that have already managed to seize the upper surface of their wing. Filmed at normal speed (~30 frames per second).
(MP4)
