# Supplementary material for: Biological and behavioral features and colonization of the sylvatic mosquito Sabethes identicus (Diptera: Culicidae)
Source: PLoS One. 2023 Dec 21;18(12):e0296289. doi: 10.1371/journal.pone.0296289 (PMC10735041; doi:10.1371/journal.pone.0296289)
Supplement: S1 Table — (DOCX) [file pone.0296289.s001.docx]

S1 Table: Daily number and percentage of emerged adults of *Sabethes identicus* in the laboratory

| Days | Cages / replicas | | | | | | | | ∑ ♂ | ∑ ♀ | Total | % |
| --- | --- | --- | --- | --- | --- | --- | --- | --- | --- | --- | --- | --- |
|  | G1 | G1 | G2 | G2 | G3 | G3 | G4 | G4 |  |  |  |  |
|  | ♂ | ♀ | ♂ | ♀ | ♂ | ♀ | ♂ | ♀ |  |  | ♂+♀ | ♂+♀ |
| 1 | 0 | 0 | 0 | 0 | 0 | 0 | 0 | 0 | 0 | 0 | 0 | 0% |
| 2 | 0 | 0 | 0 | 0 | 0 | 0 | 0 | 0 | 0 | 0 | 0 | 0% |
| 3 | 0 | 0 | 0 | 0 | 0 | 0 | 0 | 0 | 0 | 0 | 0 | 0% |
| 4 | 0 | 0 | 0 | 0 | 0 | 0 | 0 | 0 | 0 | 0 | 0 | 0% |
| 5 | 0 | 0 | 0 | 0 | 0 | 0 | 0 | 0 | 0 | 0 | 0 | 0% |
| 6 | 0 | 0 | 0 | 0 | 0 | 0 | 0 | 0 | 0 | 0 | 0 | 0% |
| 7 | 0 | 0 | 0 | 0 | 0 | 0 | 0 | 0 | 0 | 0 | 0 | 0% |
| 8 | 8 | 14 | 15 | 10 | 18 | 19 | 16 | 18 | 57 | 61 | 118 | 39,33% |
| 9 | 32 | 26 | 25 | 30 | 17 | 16 | 19 | 17 | 93 | 89 | 182 | 60,66% |
| Total | 40 | 40 | 40 | 40 | 35 | 35 | 35 | 35 | 150 | 150 | 300 | 100% |
