# Supplementary material for: Biological and behavioral features and colonization of the sylvatic mosquito Sabethes identicus (Diptera: Culicidae)
Source: PLoS One. 2023 Dec 21;18(12):e0296289. doi: 10.1371/journal.pone.0296289 (PMC10735041; doi:10.1371/journal.pone.0296289)
Supplement: S2 Table — (DOCX) [file pone.0296289.s002.docx]

**S2 Table.** Odds ratio (OR) for larva to turn into pupa in three day periods versus 15 to 17 days (baseline), according to larval density (30, 60 and 90 larvae/pan) and the luminosity condition of rearing (12 h:12 h light:dark cycle and 24h dark).

| **Variable** | **Logistic model with larval density and luminosity condition** | | | **Logistic model with larval density** | | |
| --- | --- | --- | --- | --- | --- | --- |
|  | **OR** | **CI 95%** | **p-valor *(Wald)*** | **OR** | **CI 95%** | **p-valor *(Wald)*** |
| ***18 to 19 days*** |  |  |  |  |  |  |
| Intercept | 0.704 | (0.458-1.080) | 0.108 | 0.784 | (0.556-1.105) | 0.165 |
| **Larval density** |  |  |  |  |  |  |
| 30 larvae | 2.227 | (1.012-4.901) | 0.047 | 2.233 | (1.015-4.911) | 0.046 |
| 60 larvae | 2.038 | (1.136-3.657) | 0.017 | 2.050 | (1.144-3.676) | 0.016 |
| 90 larvae | 1 | - | - | 1 | - | - |
| **Luminosity condition** |  |  |  |  |  |  |
| Dark | 1.244 | (0.743-2.085) | 0.406 |  |  |  |
| Light:Dark | 1 | - | - |  |  |  |
| ***20 to 21 days*** |  |  |  |  |  |  |
| Intercept | 0.255 | (0.142-0.460) | <0.001 | 0.270 | (0.165-0.443) | <0.001 |
| **Larval density** |  |  |  |  |  |  |
| 30 larvae | 4.618 | (1.867-11.425) | 0.001 | 4.625 | (1.870-11.440) | 0.001 |
| 60 larvae | 2.898 | (1.375-6.109) | 0.005 | 2.907 | (1.379-6.127) | 0.005 |
| 90 larvae | 1 | - | - | 1 | - | - |
| **Luminosity condition** |  |  |  |  |  |  |
| Dark | 1.124 | (0.586-2.157) | 0.724 |  |  |  |
| Light:Dark | 1 | - | - |  |  |  |
| ***22 to 40 days*** |  |  |  |  |  |  |
| Intercept | 0.115 | (0.051-0.259) | <0.001 | 0.108 | (0.052-0.224) | <0.001 |
| **Larval density** |  |  |  |  |  |  |
| 30 larvae | 7.721 | (2.539-23.479) | <0.001 | 7.708 | (2.536-23.435) | <0.001 |
| 60 larvae | 3.647 | (1.329-10.009) | 0.012 | 3.634 | (1.325-9.969) | 0.012 |
| 90 larvae | 1 | - | - | 1 | - | - |
| **Luminosity condition** |  |  |  |  |  |  |
| Dark | 0.879 | (0.380-2.035) | 0.763 |  |  |  |
| Light:Dark | 1 | - | - |  |  |  |
|  |  |  |  |  |  |  |
